# The necessary conditions of engagement for the therapeutic relationship in physiotherapy: an interpretive description study

**DOI:** 10.1186/s40945-018-0044-1

**Published:** 2018-02-17

**Authors:** Maxi Miciak, Maria Mayan, Cary Brown, Anthony S. Joyce, Douglas P. Gross

**Affiliations:** 1 Alberta Innovates, 1500, 10104 – 103 Avenue NW, Edmonton, AB T5J 0H8 Canada; 2 Faculty of Extension, University of Alberta, 10230 – Jasper Ave, Edmonton, AB T5J 4P6 Canada; 3 Department of Occupational Therapy, University of Alberta, 8205 114 Street, 2-64 Corbett Hall, Edmonton, AB T6G 2G4 Canada; 4 Department of Psychiatry, University of Alberta, 1E1 Walter Mackenzie Health Sciences Centre, 8440 112 St NW, Edmonton, AB T6G 2B7 Canada; 5 Department of Physical Therapy, University of Alberta, 8205 114 Street, 2-50 Corbett Hall, Edmonton, AB T6G 2G4 Canada

**Keywords:** Therapeutic alliance, Working alliance, Psychotherapy, Patient-therapist interaction, Patient-therapist relationship, Patient-centred care

## Abstract

**Background:**

The therapeutic relationship between patient and physiotherapist is a central component of patient-centred care and has been positively associated with better physiotherapy clinical outcomes. Despite its influence, we do not know what conditions enable a physiotherapist and patient to establish and maintain a therapeutic relationship. This knowledge has implications for how clinicians approach their interactions with patients and for the development of an assessment tool that accurately reflects the nature of the therapeutic relationship. Therefore, this study’s aim was to identify and provide in-depth descriptions of the necessary conditions of engagement of the therapeutic relationship between physiotherapists and patients.

**Methods:**

Interpretive description was the qualitative methodological orientation used to identify and describe the conditions that reflect and are practically relevant to clinical practice. Eleven physiotherapists with a minimum 5 years of clinical experience and seven adult patients with musculoskeletal disorders were purposively sampled from private practice clinics in Edmonton, Canada. The in-person, semi-structured interviews were completed in a location of the participant’s choice and were audio recorded and transcribed. Qualitative content analysis was used to analyze the textual data and constant comparison techniques were integrated to refine the categories and sub-categories. Rigour strategies used throughout the study were peer debrief, interview notes, reflexive journaling, memoing, member reflections, audit trail, and external audit.

**Results:**

Four conditions were identified as necessary for establishing a therapeutic relationship: *present*, *receptive*, *genuine*, and *committed*. These conditions represent the intentions and attitudes of physiotherapists and patients engaging in the clinical interaction. Although distinct, the conditions appear related as being present and receptive create a foundation for being genuine and committed.

**Conclusions:**

These conditions of engagement are needed for physiotherapist and patient to “be” in a therapeutic relationship. Although communication skills are important for advancing therapists’ relational abilities, awareness and integration of intentions and attitudes are essential for shaping behaviors that develop the therapeutic relationship. These findings also suggest there are characteristics of the therapeutic relationship specific to physiotherapy. Therefore, theories from other contexts (e.g., psychotherapy) should be used judiciously to guide physiotherapy practice and research.

## Background

The therapeutic relationship between patient and provider is considered a central component of patient-centred care [1, 2] and patient engagement [3, 4]. In physiotherapy, the therapeutic relationship is integrated into various practice standards [5], indicating its importance in shaping competent care. Research demonstrating a positive association between better therapeutic relationships and patient satisfaction [6], adherence with treatment [7], and clinical outcomes [8–10] supports physiotherapists’ beliefs that the therapeutic relationship influences clinical outcomes [11].

Study of the therapeutic relationship in physiotherapy is in its infancy, especially when compared to theoretical development and empirical investigation in the psychotherapy context. Despite its potential to impact clinical outcomes, we know very little about what constitutes a therapeutic relationship in physiotherapy. Due to this gap in physiotherapy literature, combined with the advanced knowledge development in psychotherapy relative to other healthcare disciplines (e.g., medicine) and the potential benefits of adopting a psychologically-informed perspective in rehabilitation [12], physiotherapy research and practice have been influenced by psychotherapy theory [9, 10, 13]. For instance*,* physiotherapy researchers have used Bordin’s theory of the working alliance [14], while educators reference Freudian [15, 16] and Rogerian principles [15]. Of these theories, Rogers’ [17] “necessary and sufficient conditions” of genuineness (freedom to be one’s self), empathic understanding (understanding of the patient’s feelings and meanings combined with congruent interactional behaviours) and unconditional positive regard (accepting attitude) have contributed, implicitly or explicitly, to the understanding of the therapeutic relationship in psychotherapy [18]. These guiding principles are broad and arguably extend to human relationships in general [17, 19] and in a way that can be understood by practitioners and patients alike. Meta-analyses have demonstrated that empathy [20] and positive regard [21] are moderately associated with clinical outcomes in psychotherapy.

While Rogers’ conditions are broad and could apply to physiotherapy as well as other healthcare disciplines [17], there might also be aspects specific to physiotherapy [22]. For instance, physiotherapists often use touch during assessment and treatment, which is likely not the case in psychotherapy-oriented disciplines, such as psychology. It is also relevant to note that delivery of physiotherapy services differs practically from other healthcare professions. For instance, physiotherapy treatment sessions can be longer in duration and occur on a more frequent basis during a particular treatment period (e.g., number of sessions per week) compared to physician visits. In addition, physiotherapists may be more likely to form consistent relationships with their patients (i.e., same therapist sees the patient) over the course of a treatment period than, for example, nurses working in a hospital where shift changes require a patient work with more than one nurse. These factors could shape how physiotherapists approach interactions with patients and create an environment that provides the opportunity to develop the therapeutic relationship as a central component of the clinical interaction, as well as direct how the therapeutic relationship should be assessed.

The concept of engagement is an influential factor in outcomes and has been linked to the therapeutic relationship. In their content analysis of patient engagement, Higgins et al. [3] determined that the therapeutic alliance (a term used broadly as synonymous with the therapeutic relationship) was an attribute of patient engagement because, as a supportive partnership, it encourages patients to engage in rehabilitation. But who is responsible for engaging the therapeutic relationship, physiotherapist or patient? For instance, Higgins et al. [3] define engagement as “.. . the desire and capability to actively choose to participate in care in a way uniquely appropriate to the individual in cooperation with a healthcare provider or institution for the purposes of maximizing outcomes or experiences of care” (p. 33). This implies a substantial degree of patient investment along side the provider.

Given the importance placed on patient engagement in rehabilitation, understanding the therapeutic relationship in physiotherapy from patient and physiotherapist perspectives is needed. Although this view is supported in research of physiotherapy services [23], historically, patient involvement in research of the therapeutic relationship has focused more on therapist perspectives. Moreover, patients’ experiences of the therapeutic relationship may have greater weight than therapists’ considering their ratings of therapeutic relationship quality can be more predictive of successful psychotherapy interventions [24]. Therefore, patient contributions are essential for developing foundational knowledge of the therapeutic relationship in physiotherapy.

Assuming meaningful engagement relies on a positive supportive relationship between patient and provider, we posed the question: what conditions are necessary for both physiotherapist and patient to engage in a therapeutic relationship? Given the nature of the question and the limited understanding of the therapeutic relationship in physiotherapy, we undertook a qualitative investigation, using physiotherapist and patient perspectives, to identify and provide in-depth descriptions of the conditions of engagement necessary for a therapeutic relationship between physiotherapist and patient.

## Methods

### Research team and reflexivity

The research team consisted of 4 clinicians (2 physiotherapists, 1 occupational therapist, and 1 psychologist), and a qualitative methodologist from human ecology. Two of the 5 researchers had significant experience using qualitative methods in health research and a third, the lead author, was completing this project as a component of her doctoral thesis and led all aspects of the study. In doing so, the lead author was informed by previous and extensive training in qualitative methods as well as meta-theoretical perspectives from critical realism [25, 26] and psychotherapeutic contextual theory [27]. The lead author also applied experience gained as a contributor on other qualitative research studies. It is also relevant to note that the lead author had post-graduate training in psychotherapy, which informed prior clinical practice as a physiotherapist as well as her interest in therapeutic relationship as a research topic. The therapeutic relationship was a central component of the clinical psychologist’s research program.

### Design

Interpretive description was the qualitative methodological orientation [28, 29] used to address the research question [28]. Grounded in naturalistic inquiry [30], interpretive description is a framework that guides researchers to maintain a path toward pragmatic versus theoretical findings when addressing clinical or applied problems. Interpretive description does not prescribe the use of a specific theoretical framework, as do traditional methods (e.g., grounded theory, phenomenology). When designing a study, Thorne suggests researchers consider various factors that could influence practice, including the disciplinary mandate (e.g., physiotherapy’s social mandate to help others), current practice theories or models (e.g., patient-centred care), and the research question. This practice-oriented scope is meant to ensure that “... at least one foot be firmly placed on the solid ground that is the ‘real world’...”(p 201) of clinical practice. For this reason, an inductive approach was taken, eliminating the use of a theoretical framework or themes at the outset of the study, including psychotherapy theories or approaches.

### Setting

The setting was private practice physiotherapy clinics in Edmonton, Canada. Reasons for situating the study in these clinics included: the notable percentage of physiotherapists working in these settings (48.2% in 2016) [31]; their community location, which provided direct and possibly greater access to physiotherapy services; and the potential that a for-profit business model could influence how much emphasis physiotherapists placed on the therapeutic relationship to build a caseload.

### Participants

Physiotherapists were eligible if they had a minimum of 5 years of clinical experience and were currently working in private practice. Adult (18–64 years of age) patients with musculoskeletal complaints were eligible if they received at minimum 3 treatment sessions and were within 12 weeks of their last session. Patients were ineligible if they had co-morbid conditions limiting their cognitive capacity or ability to communicate, neurological or systemic inflammatory conditions, or if they had received wage replacement or pain and/or suffering compensation.

### Sampling strategy and recruitment

#### Physiotherapist sampling strategy and recruitment

Purposive sampling was used to recruit 11 physiotherapists (6 female). Two authors (including the lead author) who are physiotherapists used their knowledge of the private practice community to identify physiotherapists who could provide in-depth accounts of their therapeutic relationship experiences. Administrative staff in the Department of Physical Therapy, University of Alberta sent an email invitation to therapists, directing them to contact the first author with questions or if interested in participating. Upon contact, the lead author reviewed the study information sheet with all potential participants. Three therapists did not respond to the email and 1 declined to participate after speaking with the lead author. Purposive sampling enabled sampling across factors such as treatment specializations (e.g., manual therapists) and areas of interest (e.g., chronic pain). Therapists’ ages ranged between 36 and 60 years (mean age 47.8 years); demographic data were missing for 2 therapists. All physiotherapists had been practicing in private practice for at least 10 years. The majority (10/11) used at least one advanced restricted activity (i.e., activity requiring authorization from the regulatory body), such as acupuncture or spinal manipulation [32]. Post-graduate training was reported in women’s health, vestibular rehabilitation, temporomandibular joint rehabilitation, and sports physiotherapy.

#### Patient sampling strategy and recruitment

Purposive and convenience sampling were used to recruit 7 patient participants (4 male). Ages ranged between 18 and 62 years (mean age of 42.3 years). Administrative staff in 3 clinics purposively identified patients they believed would be able to provide candid accounts of the relationships with their therapists. Staff provided patients with study information sheets and directed them to contact the lead author with questions or if interested in participating. Study information was also distributed to a large athletic club via a coach’s email, with instructions to contact the lead author. Upon contact, the study information sheet was reviewed with all potential participants. One patient was deemed ineligible for the study after speaking with the lead author. Most patients (6/7) had previously accessed physiotherapy services and most (5/7) had experienced their physical issues for greater than 3 months prior to seeking treatment.

### Data generation and analysis

Data generation and analysis were inductive and iterative. After receiving informed consent, semi-structured one-on-one interviews were completed in a public location of the participant’s choice, audio-recorded, and professionally transcribed. One interview lasting between 40 and 90 min was completed with each participant, although participants were informed they may be contacted to clarify their statements. An interview guide [33] of open-ended questions was used to facilitate descriptions of participants’ experiences of the therapeutic relationship. Although physiotherapists and patients had separate interview guides, they were similar in that both began with broad questions to provoke responses on the clinical interaction in general (e.g., What do you call yourself – a patient or a client?) then became specific to aspects of the interaction that physiotherapists and patients believed could influence or were a part of the therapeutic relationship. However, questions in the interview guides differed since physiotherapists form therapeutic relationships with many patients whereas patients will not have this breadth of experience. For example, physiotherapists were asked about their views on ‘fixing patients’ in order to encourage responses regarding their treatment philosophies in general whereas patients could be asked to compare their therapeutic relationship with their physiotherapist to the one with their physician. We have described the rationale for both patient and physiotherapist interview guides elsewhere [34]. Probing questions (e.g., How did that make you feel? or What happened then?) or contact statements to check for clarity (e.g., It sounds like your physiotherapist was concerned about your well-being?) were used to build on participant responses in-the-moment to encourage thorough description and to disrupt the researcher’s pre-conceived notions. Various rigour strategies, described below, were used to critique the data generation process in order to continually inform interview quality. For example, interview notes were a component of the lead author’s intersubjective reflection on the “... situated, emergent, and negotiated nature of the research encounter” ( [35] p8). Interview notes were completed after each interview to capture the researcher’s impressions and critique of, for example, the interview setting as well as the interaction between researcher and participant, including how the researcher’s perspectives on the therapeutic relationship might have influenced the interview. Two mock interviews were completed, which informed refinement of the interview guides prior to initiating participant interviews. Concurrent data generation and analysis allowed for interview guide revisions to reflect the evolving analysis. The lead author completed all interviews and data analysis. Data were generated until a point of saturation [36] was achieved representing a meaningful reflection of clinical reality.

Data analysis occurred in 2 concurrent phases: (1) a systematic process of data (audio and transcript) review, reflexive journaling, and memoing prior to coding; and (2) formal coding guided by qualitative content analysis [37] and constant comparison principles [38]. To support an inductive process that would generate findings congruent with the physiotherapy context, psychotherapy theory (e.g., Rogerian theory) was not used to guide the analysis. Content analysis began with initial coding [38] or the assignment of a specific word or phrase to summarize a key attribute of a portion of text [39]. As patterns of codes were recognized [40, 41], they were grouped into categories and sub-categories [42]. At this point, constant comparison strategies were integrated to refine the analysis and assist in the process of thinking about the categories’ properties (i.e., characteristics of the category) and conditions (i.e., circumstances that foster the category) [36]. Negative cases [43] within participant accounts contributed to clarifying aspects of the conditions of engagement.

The lead author completed all interviews and analysis in partial fulfillment of her doctoral thesis. It is worth noting that the lead author had not met the patient participants prior to the study. However, given the lead author had previously worked in private practice physiotherapy, she knew some of the physiotherapist participants on a professional basis, to varying degrees, prior to the study commencing. Various rigour strategies that involved researcher, participants, and external reviews were used throughout the study to address transparency and trustworthiness of the research process and findings. Personal researcher strategies involved journaling to: maintain an audit trail [44, 45]; reflexively engage [46] throughout the research process; and memo questions and ideas during the analysis [47]. Two patient participants engaged in member reflections [45] about the ongoing analysis and 2 researchers and healthcare providers were involved in peer debrief [44, 47]. An external audit [44] was completed at project completion, confirming that the research process was thorough and the quality and nature of the findings were congruent with the process. NVivo 10 for Windows software (QSR International Pty Ltd.) was used to manage the data and analysis.

## Results

Four foundational conditions fostering engagement between physiotherapist (PT) and patient within a therapeutic relationship were identified and labeled: (a) Present, (b) Receptive, (b) Genuine, and (d) Committed.

### Present

Being present reflects physiotherapists’ and patients’ intentions and abilities to be in-the-moment or embodied in time and space. Physiotherapists make conscious choices about the amount of time they spend in direct proximity with patients in a potentially chaotic setting laden with competing responsibilities. Therapists described instances when remaining with the patient was believed to be of utmost importance, such as when a patient needed “more one-on-one time” (PT-J) for guidance with exercises or when experiencing emotional distress:

PT-B:... they start crying. .. the biggest thing. .. is don’t pull away. Don’t walk out of the room. Don’t leave them.

While scheduling longer sessions (e.g., 30 min) was an option, physiotherapists also described many impromptu situations where a decision was made to remain with a patient, despite the allotted timeframe:

PT-I: I think that if I’m with somebody who’s gone through 20 years of struggle with this, I think I have to take more time at the beginning.

Patients noticed their therapists’ efforts to “spend more time with me than they should” (Patient-B). Patient-E appreciated that “time was of no consequence” because it gave the impression that the therapist was willing to do “whatever it takes” to address the issue. Patients also noticed when therapists were not present and the negative impact this had on their experiences, such as when they perceived therapists were rushing. Moreover, patients were able to distinguish between a ‘busy’ therapist and a ‘rushed’ therapist, where a busy therapist could be present despite the hectic environment:

Patient-D: They were busy as can be, just on a cycle going from one to the next to the next and coming back. They always took the time to make you feel like you were a decent person.

In addition, physiotherapists and patients described the importance of creating a “bubble” (PT-K) that allows full engagement. Although therapists could be distracted by multiple responsibilities, a busy caseload, and personal factors (e.g., family stressors), they took personal responsibility to “turn those issues off” (PT-G) when with patients. Therapists also described using non-verbal cues and manipulating material space, such as adjusting seating arrangements and using private rooms versus curtained cubicles to help patient and therapist “narrow down” (PT-E). Patients also spoke of their need to be present during the interaction. Notably, they spoke of being in-the-moment to understand their bodies and “feel the treatment” (Patient-E) because “if I can’t tell her [PT] how it’s feeling or how it’s reacting, I can’t help her” (Patient-A).

### Receptive

To be receptive, physiotherapists and patients must enter interactions with: a) an *open attitude* to negotiate appropriate treatment plans; and b) a *focused receptivity* to identify salient issues and needs.

#### Open attitude

Having an open attitude requires physiotherapists and patients to manage personal agendas and be willing to be “open to all these things [treatments]” (Patient-A). Even though therapists have specific knowledge and skills that inform treatment plans, they also need “... to be open and listening and not go into this [interaction] with a pre-determined agenda”(PT-B). This includes a willingness to listen to the patient’s story because it is “... important to me as the patient that you hear and understand what I need you to help me [with]” (Patient-E). Allowing patients to tell their stories can be important for developing a safe and receptive atmosphere:

PT-I: The big thing is that patients that are struggling and... really have big problems, they need to tell their story. You need to listen and shut your mouth.

The same is true for patients. Just as therapists need to “... listen to all their [patients’] fears, all their issues...” (PT-G), to create a working relationship, patients also need to listen and be open to physiotherapists’ suggestions:

PT-G: You try to explain what you are doing and they keep interrupting you. They keep challenging everything you say... They don’t listen to anything you say. That I find really difficult.

#### Focused receptivity

In addition to an *open attitude*, physiotherapists must also be attentive to the situation at hand. This is achieved by actively considering patients’ verbal and non-verbal cues. For example, focused receptivity helps therapists gain insight into patients’ physical and psychological states:

PT-B: They are guarded, they are tightening. .. you can just see that they are upset.

PT-A: If they are not talking to you. .. or if their tone has raised or heightened then you know something is going on...

In addition to focusing on behaviours, therapists also spoke of how being receptive to patients’ comments, often noted either mentally or in the chart, was essential for identifying how to connect with patients about their lives. This enabled them to “... gauge where that person’s at and what their interests are...” (PT-E). This receptivity fosters deeper engagement during the immediate interaction and provides opportunity for the same in the future.

### Genuine

To be genuine is to be real or convey sincerity in the present. Being genuine in a therapeutic relationship has three aspects: a) *being yourself;* b) *being honest;* and c) *investing in the personal*.

#### Being yourself

To convey genuineness, individuals must remain congruent with their personal qualities and values, while maintaining an accepting attitude. To do this, physiotherapists and patients must feel comfortable enough to sincerely present themselves, not putting on a facade:

PT-I: I’m pretty open with people. I can talk to anybody... I don’t change who I am in any role in my life. .. I am who I am. I think patients probably feel comfortable asking me that because that’s kind of how we interact as people.

Patients notice when physiotherapists are being themselves or have “warm”, “personable”, or “approachable” personalities. In doing so, therapists create an environment where patients can also express themselves. Therapists curb judgment of patients and are open to “where that individual is” (PT-E) by acknowledging their unique personalities, life stories, and social and cultural realities. In addition, freedom for patients to be themselves extends to their bodies and injuries. Physiotherapists can mitigate patients’ feelings of vulnerability that give rise to negative perceptions of their bodies and injuries:

Patient-D:... [he] was very good at making me feel like you weren’t abnormal... I don’t want to be singled out as out of shape or old or... I didn’t quite know what to expect when the physiotherapist came in... I expected a fair bit of judgmenty-type things the way that doctors would sometimes.

#### Being honest

While honesty is likely a necessary condition for any healthy relationship, there are two main qualities that describe being honest in the physiotherapy context: *transparency* and *directness*. Being *transparent* involves therapists and patients providing the necessary information to help the patient progress in a safe and meaningful way. This can include impressions of the physical problem and rehabilitation process; personal limitations in skill and knowledge; patient participation and outcome expectations; and the therapist’s role and responsibilities:

PT-B:... being realistic about what’s going to happen. .. I’m really honest with people about that and I explain to them and especially with those more complex, that they are 80% of what’s going to make a difference.

Patients must also be transparent about information related to their conditions, or as Patient-C claims, it “... is important for the patient to tell the whole truth...” Physiotherapists agreed they needed to trust that “... they [patients] are telling you the truth... all the factors that are contributing.” (PT-E).

In addition to being transparent, the physiotherapist must also be *direct* in the tone and manner of communication. Specifically, therapists must be clear and forthright. Although being direct might be interpreted as stern, especially in challenging situations, the tone can also convey concern or compassion. Ultimately, the therapist’s intention is to be clear, leaving little doubt about the message:

PT-H: She did have an injury but I had to explain to her that, “The injury that you have cannot cause all of the problems that you are having. Let’s try to figure out what else is causing it.”

#### Investing in the personal

A primary focus of physiotherapy is to restore or maintain physical mobility and function. However, many patients and physiotherapists revealed that a personal aspect was important to the overall quality of the therapeutic relationship. Being invested in the personal was revealed through *an interest in the person* and *a willingness to disclose* about self.

Taking *an interest in the person* pertains to therapists’ or patients’ desires to broaden the scope of caring to an interest in the other’s life beyond the reason for referral:

PT-C:... folks that ask me how I’m doing, folks that ask me how things are going, we end up talking about things unrelated to their condition or the weather... We have an interest in each other.

Even though therapists often need to know about patients’ lives for therapeutic reasons, those invested in the personal are willing to get to know the patient as a person, demonstrating an authentic interest in people’s lives. This investment can put the patient at ease:

PT-I: Even when my questioning starts, you know I always ask them about them first. So, I always make it clear that that’s really important to me... I ask them to tell me a little bit about yourself outside of what’s brought you here... What sorts of things do you enjoy doing? Even the way I ask those questions is very different. ... I can get to a person’s level of comfort and they can relax a little bit if I ask them questions that are not directed to their sore knee or sore shoulder. . .

Even though roles and professional boundaries might make it difficult for patients to express an interest in their physiotherapists’ lives, they could be “genuinely interested” in getting to know their therapists, asking “... almost as many questions as you ask them” (PT-J). Furthermore, some patients found value in knowing their physiotherapists on a human level:

Patient-B: It makes a huge difference knowing that they can relate to you, first of all and they have a real life. They are not just a physio... these people go home and have kids and have a family. It’s nice. You are both real people so you should probably treat each other like people.

Another aspect of investing in the personal is demonstrating *a willingness to disclose*. Being willing to disclose means offering something more personal and not necessarily related to the primary intent of the interaction. Therefore, disclosures can be social or therapeutic. Most therapists recalled they had different perceptions of what constituted an appropriate disclosure:

PT-F: ... you can talk about personal interests and not get personal so hobbies and what you might do in your non-professional life that doesn’t have to do with anything intimate... sports are good, music is good, leisure activities. . .

Patients’ investments in the personal also included disclosing more personal aspects of their physical and emotional challenges, including issues pertaining to sexuality or mood. Although one therapist commented that there are some patients who “... are comfortable disclosing that information to you” (PT-A), this same therapist also claimed that patient disclosures sometimes required a “leap of faith” in the therapist. Patients agreed, commenting that disclosure of their physical issues and personal lives was easier as “... you get more comfortable so you’re more willing to tell them what you are feeling” (Patient-C).

**Fig. 1:**
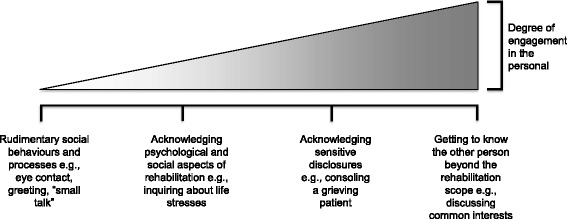
Spectrum of Personal Engagement. The figure illustrates personal engagement as a spectrum involving a relationship between the nature of engagement and the degree of personal engagement. The degree of personal engagement is dependent on the intentions and behaviours of physiotherapist and patient

There is a spectrum of how much physiotherapists and patients are willing to invest in the personal (see Fig. 1). For example, PT-D was very clear he was not interested in his patients’ personal lives, making “... a point to stay outside of those kinds of conversations”, nor was he interested in discussing anything outside of the clinical problem:

PT-D: I really don’t talk much on the personal side. I really don’t think any of my patients even know how many kids I have or what I do in my spare time. I don’t think any one of them knows that... that’s purely on the personal side.

Therefore, it appears there are different ways to be in a therapeutic relationship:

PT-K: My partner is exactly the opposite of me. .. my professional boundaries and his professional boundaries are on either side of the continuum of professional boundaries.

### Committed

To be engaged, physiotherapists and patients must be committed to their roles within the therapeutic relationship. A patient’s well-being matters, or, as PT-A claimed, “their well-being is your well-being...” This speaks to an ethic of care that encompasses physiotherapists’ professional duty and the desire to be of service to others to restore patients’ well-being. Some physiotherapists and patients stated that therapists do not “fix” patients, but that both have roles they must commit to:

Patient-B: You have to take care of yourself in order for them [physiotherapists] to be able to take care of you too. If you are just going to go and expect them to do it all for you, it’s not going to happen. You’re not going to get better, I find.

These points considered, there are two aspects that characterize being committed: (a) *committed to understanding* and (b) *committed to action*.

#### Committed to understanding

Both physiotherapists and patients must be motivated to understand the patient’s situation. When the physiotherapist is committed to understanding the patient, there is a “... need to understand more about what you [patient] are describing...”(PT-B)*.* Therapists were not satisfied with a generic overview of the patient’s situation:

PT-D:. .. if you give out the impression that you know what’s happening in this person’s back without showing them the interest or without making an effort in understanding it, you won’t be able to help them.

The physiotherapist is not only dedicated to understanding the patient’s physical situation, but also “a picture of the unspoken” (PT-C) or the psychosocial factors that could be influential:

PT-H: If a person has what we would call a chip on their shoulder let’s say, you try to find out what the chip is. I see it as part of my job to get over that chip... If I can find out what brought it on... Empathize. Sort of understand.

Even though the physiotherapist is expected to try to understand, it was also clear that patients needed to invest in understanding their situations:

Patient-E: I felt I needed to understand as much of my own physiology and biology in order to help what it is that she was trying to do for me, so I could help myself.

#### Committed to action

Being committed to action involves making “all efforts” (PT-D) to honour the best interests of the patient. Physiotherapists “... do their best to do the best that they can...” (Patient-C), and will go beyond due diligence to help patients achieve goals. Therapists committed to action recognize there are many facets of care to be considered, and that they may need to “go that extra little mile” (PT-A) in complicated situations.

Patients must also be committed to act in their own best interests. Physiotherapists spoke about the necessity of patient “buy in” or as PT-G stated, “... they also have to agree with what you are saying and be motivated to take part in the treatment themselves because it's not just passive.” Patients seemed to understand that their motivation to participate was essential:

Patient-G:... they [patients] are expecting the physiotherapist to “fix them” and they don’t need to fix themselves... I understand what physio means and how I need to aid myself as well.

Patients highlighted that continuity, described as the patient seeing the same therapist versus being shuttled between therapists, is an important part of being committed. Having “your therapist” (Patient-B) facilitates progression of the session, reduces the need for the patient to familiarize a new therapist, and allows the physiotherapist to get to know the patient’s body, activity levels, and treatment history:

Patient-G: “What’s your past injuries? How many injuries have you had? What's your sport history?” All that stuff. When I saw (name of physiotherapist), it was like, “Oh hey (name of patient). What do we need to work on today?” He already knows how much I exercise and everything.

#### The Conditions of Engagement Form a Safe Therapeutic Container

**Fig. 2:**
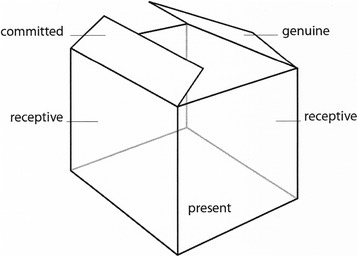
The Safe Therapeutic Container Formed by the Conditions of Engagement. The foundation and the walls of the therapeutic container represent the two cornerstone conditions, “present” and “receptive”, respectively. “Committed” and “genuine” are more variable and are therefore represented by the mobile nature of the lids of the container

The conditions of engagement work in concert to form a safe therapeutic container for the therapeutic relationship to manifest (Fig. 2). The foundational components of the container – the bottom and the walls – are represented by the cornerstone conditions *being present* and *being receptive. Being present* is the foundation that allows the other conditions to unfold, while *being receptive* provides the structure that enables pertinent information to be gathered. There is more of a personal aspect to *being genuine* and *being committed*; the degree to which either condition is established is reliant upon individuals’ uniqueness and circumstances. Essentially, the conditions of engagement set the tone for “being” with other and self, representing the dynamic intent to engage that both physiotherapist and patient bring to the relationship.

## Discussion

We found there are necessary conditions of engagement that facilitate the therapeutic relationship in physiotherapy. In addition to providing needed clarity specific to physiotherapy, these conditions offer insight into the relevance of psychotherapeutic principles in physiotherapy and how they can best serve practice and research.

### The Relevance of Psychotherapeutic Principles in Physiotherapy

Physiotherapy has previously borrowed theory from psychotherapy to inform research and practice. In fact, physiotherapy can be seen to clearly align with the elements of a psychotherapeutic process that involves an individual seeking healing (e.g., patient) and a healing agent (e.g., physiotherapist) investing in a relationship in order to relieve disability and suffering while addressing the individual’s beliefs and attitudes [27, 48]. This is a compelling perspective as researchers and clinicians turn their attention to psychological aspects rehabilitation, such as patient expectations, beliefs, and emotions alongside addressing physical impairment and function [12, 48]. Given our results appear to have similarities with Rogers’ necessary conditions of patient-centred care [49], and that these principles inform motivational interviewing [50], an intervention increasingly being used in healthcare settings with positive results [50–53], we feel there is an opportunity to consider their relevance in physiotherapy. This is also important considering the pragmatic differences between physiotherapy and psychotherapy, including the conditions (e.g., mental illness versus physical conditions) [12] and the subsequent treatment goals.

Aspects of Rogers’ genuineness, unconditional positive regard, and empathic understanding weave into our conditions of engagement. For instance, Rogers describes genuineness as being the expression of an integrated self through self-awareness and transparency [18], which is congruent with our description of genuine as physiotherapists honouring their personal psychosocial situations, disclosing personal information, and being direct with patients.

While our findings complement Rogers’ conditions, clear nuances are also present. Specifically, we added being *present* and *receptive*. Rogers [17] limits his discussion of being present to the basic level of patient and therapist being “in contact” (p. 90), and to some degree influencing the experience of the other. However, we define being present as a foundational condition and clearly describe the focused manner and intentional use of time and space in creating a safe therapeutic environment. In addition, we explicitly identify *receptive* as a condition. Although some might interpret being receptive as an aspect of Rogers’ empathic understanding, we understand it to have distinguishing characteristics, namely that a therapist can be receptive but not be empathic.

One difference between Rogers’ and our conditions relates to who is responsible for developing the conditions of engagement. Rogers describes the psychotherapist as cultivating the conditions of engagement whereas our findings indicate both physiotherapists and patients must contribute. We agree that the practitioner is responsible for establishing the conditions that provide a safe space for the patient to engage. Indeed, the physiotherapist’s capacity to do so could be the deciding factor in some patients’ willingness to engage. However, our participants were clear that engagement involves the deliberate participation of both patient and therapist for the conditions to flourish. This is consistent with Bright et al.’s [4] concept analysis of engagement in rehabilitation, which concluded that both clinicians and patients have roles in patient engagement. Lequerica et al. [54] also found that therapists’ ability to facilitate patient engagement was supported by “... taking time to simply talk to the patient about their life...” (p. 757), indicating that engagement is two-way and that both therapist and patient engagement can be essential in developing the conditions.

### The Impact of Conditions of Engagement on Physiotherapy Research and Practice

Psychotherapeutic theories such as Rogers’ conditions [17] and Bordin’s working alliance [14] are claimed to be universal. Moreover, physiotherapy researchers tend to assume that these theories directly transfer to physiotherapy. By clarifying physiotherapy-specific conditions of engagement, our findings clearly have the potential to impact physiotherapy research and practice.

Regarding research, we need to consider whether measurement scales developed through a psychotherapeutic lens are valid within the physiotherapy context. This view is congruent with Besley et al.’s conceptual [22] and evaluative [55] findings. In particular, the evaluative findings clarify that while the measurement properties for the Working Alliance Inventory [56] and Helping Alliance Questionnaire [57] were “adequate” [55], there were also aspects missing. The authors called for a better conceptual understanding within the physiotherapy context in order to develop more rigorous measurement tools.

Regarding practice, the conditions of engagement speak to the essence of what is required to have a meaningful therapeutic relationship. Much literature has focused on the importance of communication in developing the therapeutic relationship [58]. However, relationships are more than a compilation of skills and behaviours that can be dutifully checked off when completed. Relationships are dynamic, requiring intent to ensure behaviours and skills are congruent with the situation. Additionally, it is important to note that a personal aspect can be important for physiotherapy therapeutic relationship. Even though participants described a spectrum of perspectives and practices regarding the nature and boundaries of the personal, the majority agreed that a personal aspect, understood as patients’ and therapists’ authentic interest in the other’s life outside of the rehabilitation context or the disclosure of information perceived as private, was important. This study illustrates that patients and therapists may want to know one another as people while respecting professional boundaries. Moreover, the conditions provide the foundation for a patient-centred approach to be operationalized in clinical practice. Being receptive, committed, and genuine create the safe therapeutic space necessary for a patient-centred exchange that highlights collaboration in order to establish meaningful patient-driven goals [1].

As alluded to above, it is worth noting that the conditions supporting the therapeutic relationship do not ‘just happen’ by completing a list of behaviours. The conditions in this context exist, at least in part, as a function of physiotherapist and patient states or the quality of consciousness experienced by an individual in any given moment, whereas a condition of engagement can be described as the sentiment or circumstances between two individuals. An individual’s state is informed by a complex merging of momentary thoughts, feelings, and sensations in addition to more enduring attitudes, values, and beliefs, which will inform that individual’s intentions and ability to behave in ways that carry out those intentions. Moreover, behaviours that are genuine and congruent with a situation arise from appropriate states. Therefore, if physiotherapists are aware of and able to critique their thoughts, emotions, attitudes and assumptions [59] and adjust as needed, conditions can be developed, maintained, or deepened. This reflection can occur outside of the clinical interaction, which is more likely with novice physiotherapists or within the clinical interaction, otherwise known as ‘reflection-in-action’ [59]. Reflective practice targeting therapeutic relationships is critical for encouraging physiotherapists’ abilities to cultivate the conditions of engagement. Without it, physiotherapists risk self-limiting their ability to influence what is considered a key contextual factor [48] impacting clinical outcomes [8–10].

### Limitations of the study

There are three main limitations in this study. First, both patient and physiotherapist accounts often centred on therapists’ contributions to the therapeutic relationship and conditions of engagement. Although this might be expected given the therapist’s role and position of power within the clinical interaction, a second interview with patients would have provided opportunity to probe them about their role in establishing the conditions of engagement. Second, the exclusion of some patients limited the nature of the data and hence, the possible breadth of the findings. Other populations likely have additional considerations (e.g., long term therapy and family involvement for patients with neurological conditions) that require focused investigation beyond the scope of this study. Third, these findings would likely be most applicable for therapists in private practice physiotherapy. Future research in other settings (e.g., hospitals, rehabilitation centres) and systems (e.g., workers compensation) will contribute to understanding the conditions that influence patients’ and physiotherapists’ abilities to engage in the therapeutic relationship. However, because the conditions of engagement are conceptual in nature, they could be useful across a wide range of physiotherapy contexts and health care professions as a foundational starting point regardless of practice area.

## Conclusions

Participants in this study have made it clear that therapeutic relationships do not ‘just happen’. Through participants’ candid accounts we have highlighted that conditions specific to the physiotherapy encounter create a safe environment and facilitate mutual engagement of therapist and patient. Cultivating these conditions, in conjunction with applying communication skills (e.g., active listening), will result in situation-appropriate responses. Findings suggest that theories developed in other disciplinary contexts (e.g., psychotherapy) should be used judiciously when developing theory that guides physiotherapy practice and research regarding the therapeutic relationship.

## Abbreviation

PT: physiotherapist
